# Patterns of muscle coordination during dynamic glenohumeral joint elevation: An EMG study

**DOI:** 10.1371/journal.pone.0211800

**Published:** 2019-02-08

**Authors:** David H. Hawkes, Omid A. Khaiyat, Anthony J. Howard, Graham J. Kemp, Simon P. Frostick

**Affiliations:** 1 Musculoskeletal Science Research Group, Institute of Translational Medicine, University of Liverpool, Liverpool, United Kingdom; 2 School of Health Sciences, Liverpool Hope University, Liverpool, United Kingdom; 3 Academic Department of Trauma and Orthopaedic Surgery, Leeds General Infirmary, Leeds, United Kingdom; 4 Department of Musculoskeletal Biology, Institute of Ageing and Chronic Disease, University of Liverpool, Liverpool, United Kingdom; Mayo Clinic Minnesota, UNITED STATES

## Abstract

The shoulder relies heavily on coordinated muscle activity for normal function owing to its limited osseous constraint. However, previous studies have failed to examine the sophisticated interrelationship between all muscles. It is essential for these normal relationships to be defined as a basis for understanding pathology. Therefore, the primary aim of the study was to investigate shoulder inter-muscular coordination during different planes of shoulder elevation. Twenty healthy subjects were included. Electromyography was recorded from 14 shoulder girdle muscles as subjects performed shoulder flexion, scapula plane elevation, abduction and extension. Cross-correlation was used to examine the coordination between different muscles and muscle groups. Significantly higher coordination existed between the rotator cuff and deltoid muscle groups during the initial (Pearson Correlation Coefficient (PCC) = 0.79) and final (PCC = 0.74) stages of shoulder elevation compared to the mid-range (PCC = 0.34) (p = 0.020–0.035). Coordination between the deltoid and a functional adducting group comprising the latissimus dorsi and teres major was particularly high (PCC = 0.89) during early shoulder elevation. The destabilising force of the deltoid, during the initial stage of shoulder elevation, is balanced by the coordinated activity of the rotator cuff, latissimus dorsi and teres major. Stability requirements are lower during the mid-range of elevation. At the end-range of movement the demand for muscular stability again increases and higher coordination is seen between the deltoid and rotator cuff muscle groups. It is proposed that by appreciating the sophistication of normal shoulder function targeted evidence-based rehabilitation strategies for conditions such as subacromial impingement syndrome or shoulder instability can be developed.

## Introduction

Shoulder pathology, such as subacromial impingement syndrome or shoulder instability, is prevalent and has a substantial impact on patient’s quality of life [1, 2]. Normal shoulder function, essential for many activities of daily living, requires the integration of strength, range of motion and muscular endurance. Understanding this is the basis for understanding pathology.

The glenohumeral (GH) joint is a multi-axial ball-and-socket synovial joint which, in combination with the acromioclavicular, sternoclavicular and scapulothoracic articulations, facilitates movement of the upper limb. Its minimal bony constraint permits a remarkable range of motion but results in translation of the humeral head on the glenoid fossa following contraction of the powerful shoulder girdle muscles [3, 4]. It has long been recognised that the rotator cuff muscles are crucial in limiting this translation and maintaining GH joint stability [5–7]: their contraction ‘stiffens’ the joint, establishing a stable fulcrum for arm movement [8]. However, more recently coordinated activity of all the major shoulder muscle groups during a functional shoulder elevation task has been shown, implying a wider muscular contribution to stability [9].

Muscular force generation must ensure a stable GH fulcrum whilst permitting the necessary net torque required to achieve movement. Inadequate stabilisation results in excessive translation of the humeral head on the glenoid fossa, which can lead to symptomatic subluxation or dislocation. Conversely, an over-stabilised system wastes energy [10]. The shoulder is particularly reliant on coordinated or balanced muscular activity to ensure that the humeral head remains centred on the glenoid fossa. The destabilising effects of the prime moving muscles are balanced by the synchronous activity of muscles whose anatomical arrangement means they can exert a stabilising effect for that particular movement. Indeed, aberrant muscle coordination is proposed to have a central role in the development of atraumatic shoulder instability [11] and shoulder impingement syndrome [12]. A full understanding of normal shoulder muscular coordination is essential as a basis to understand these pathologies and to develop effective evidence-based treatment strategies.

Muscle coordination is dynamic, as stability requirements vary according to task-specific demands [13]. The coordination between muscles can also change during a movement as the resultant force vector about the joint evolves [14]. Inherent muscle redundancy allows re-distribution of activity among the shoulder complex, which will further alter inter-muscular coordination [15]. These dynamic relationships illustrate the sophistication of normal shoulder function and current research strategies must reflect this.

Several studies have described muscle activation during single planar shoulder movements [6, 16–19]. However these principally focus on the level of muscle activation. Less attention has been given to the important concept of muscle coordination and we are not aware of any previous reports describing muscle coordination of the entire shoulder girdle and how this might vary dynamically during movement progression. This is a significant deficiency within the literature given the reliance of the shoulder on coordinated muscle activity, and the role in which aberrant coordination may play in the development of shoulder pathology.

The primary aim of this study, therefore, is to use EMG to define muscle activation patterns and dynamic inter-muscular coordination in healthy subjects during multi-planar shoulder elevation.

## Methods

### Participants

The results of 20 healthy volunteers, with no history of upper limb symptoms and no abnormality on clinical examination, are reported. The study group comprised 10 males and 10 females. Mean age was 28.4±8.5 years, mean height 1.70±0.06m and mean mass 68.6±11.1kg. The study had Local Research Ethics Committee ((NRES Committee North West–Liverpool Central) approval and informed written consent was obtained from all participants.

### EMG measurement

EMG signals were recorded using a TeleMyo 2400 G2 Telemetry System (Noraxon Inc., Arizona, USA) during the testing protocol described below. MyoResearch XP (Noraxon Inc., Arizon, USA) was used for signal acquisition and off-line analysis. Bipolar, disposable, self-adhesive pre-gelled Ag/AgCl dual surface electrodes (Noraxon Inc., Arizona, USA) were used to record the activity of the anterior deltoid (AD), middle deltoid (MD), posterior deltoid (PD), upper trapezius (UT), middle trapezius (MT), lower trapezius (LT), serratus anterior (SA), teres major (TM), latissimus dorsi (LD) and pectoralis major (PM). The skin was prepared with an abrasive paste (Nuprep: Weaver and Company, Aurora, CO, USA). Electrodes were placed in parallel to the muscles fibres according to accepted anatomical locations [20–22]. The size of the surface electrodes (conducting area 10 mm diameter; inter-electrode distance 20 mm) conformed to international guidance. The use of appropriately sized electrodes placed using anatomical criteria accepted in the literature limited the impact of cross-talk. A reference electrode was positioned on the acromion. Bipolar disposable hook wire electrodes (Nicolet Biomedical, Division of VIASYS, Madison, USA) were inserted aseptically for the intra-muscular recording of the supraspinatus (SSP), infraspinatus (ISP), subscapularis (SUBS) and rhomboid major (RM) [23, 24].

Pre-amplified leads differentially amplified the detected signal (common mode rejection ratio (CMRR) >100dB; input impedance >100Mohm; gain 500dB). The detected signals were digitalised (sampling rate 3000Hz) and band pass filtered ([10 …500]Hz for surface electrodes and [10 …1500]Hz for fine wire electrodes). EMG data acquisition conformed to international standards [25]. Manual muscle testing confirmed correct electrode placement and signals were excluded if they were of poor quality.

### EMG testing protocol

**Fig 1:**
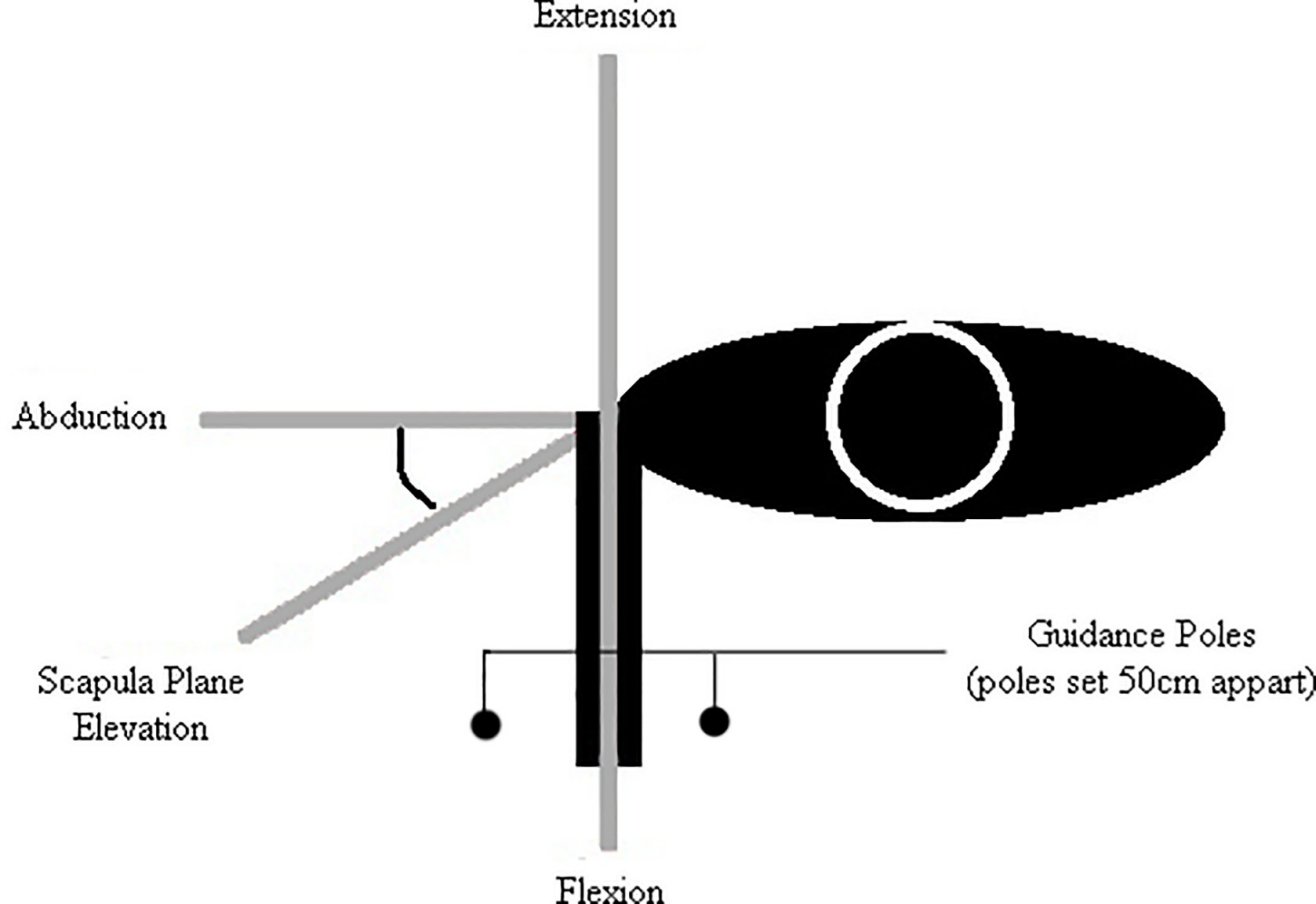
Overhead schematic of the testing protocol indicating the different planes of movement studied.

The testing protocol was performed with participants standing on a wooden board with their elbow extended, forearm in neutral rotation and their feet shoulder-width apart. Pre-marked axes were drawn on the board to guide shoulder movement between two guidance poles (50cm apart) in 4 different planes: flexion (sagittal plane movement); scapular plane elevation (movement 30^o^ anterior to the coronal plane); abduction (coronal plane movement); extension (sagittal plane movement) (Fig 1). Ten continuous cycles of maximal shoulder elevation and depression were performed in each movement plane. Participants were instructed to move in a smooth and continuous manner. The pace was governed by a metronome set at 1 beat every 2 seconds (s) such that each cycle took 4s to complete (2s for elevation and 2s for depression). The movement was performed between guidance poles to ensure that subjects maintained the correct plane. An opportunity for practice prior to EMG recording ensured familiarity with the movement task.

### Data management and statistical analysis

Recorded EMG signals were processed off-line. ECG contamination was removed using an adaptive cancellation algorithm pre-built with the MyoResearch XP software. Recorded signals were smoothed using the root-mean-square (window 100 ms). The amplitude was normalised to the mean of the recorded signal during flexion. Normalisation with respect to flexion ensured that muscle activity could be compared across the different movement planes.

This normalisation method has previously been accepted with the literature as a technique to compare relative (rather than absolute) differences in activity levels between movements [26, 27]. Amplitude normalisation to the mean was performed for two reasons. First, the primary aim of this work was to examine inter-muscular coordination, which requires the study of muscle activation patterns rather than absolute amplitudes. Second, whilst normalisation to a maximal voluntary contraction (MVC) is popular, using an isometric contraction to normalise EMG data obtained during a dynamic task may be inappropriate, as an MVC might not represent the maximum activation of the muscle at lengths other than those at which the MVC was performed [28–30].

Time normalisation was performed to allow ensemble (synchronous) averaging of the EMG data from the 10 movement cycles, thus allowing point-to-point comparison of activity. Shoulder elevation (100 periods) and depression (100 periods) were normalised individually to eliminate the problem of inter-subject variations in kinematic patterns and ensure consistent comparisons. Data for the muscle groups were calculated by ensemble-averaging the activity of the individual component muscles: deltoid (AD, MD, PD); rotator cuff (SSP, ISP, SUBS); SA/UT (SA, UT); MT/LT (MT and LT) and LD/TM (LD and TM). The deltoid muscles were combined as all accepted prime movers during shoulder elevation. The rotator cuff muscles were combined as they are predominantly described in the literature as humeral head stabilisers. The LD/TM were combined as both exert an adducting moment at the GH joint, the MT/LT as they adduct and depress the scapula, and the SA/UT as they elevate and upwardly rotate the scapula. It is acknowledged that that combining muscles into groups is artificial and an oversimplification as the rationale given above does not consider direction specific activation [31, 32]. However, it was not felt possible to simultaneously conceptualise all permutations of individual muscle combinations. Strategies to simplify the data set therefore had to be sought.

The mean signal amplitude was calculated in addition to the timing of peak muscle amplitude. Time of peak amplitude was chosen as a parameter for muscle sequencing over an onset-offset analysis due to the continuous nature of the movement protocol. Results are expressed as a mean ± SD or standard error of the mean (SEM) as appropriate. A paired t-test was used to compare amplitude during arm elevation and depression for each movement plane. A repeated measures analysis of variance (ANOVA) was used to compare muscle activity and timing across the different movement planes. A p value of ≤0.05 was accepted as significant.

Cross correlation is an established method for comparing EMG signals and was used to quantify the coordination between different muscles and muscle groups [33, 34]. The cross-correlation coefficient is a measure of the similarity in shape between two curves, being sensitive in general to similarities and differences in temporal characteristics and when overall timing is similar to similarities and differences in shape. The Pearson Correlation Coefficient (PCC) normalises the covariance of two signals with respect to the product of the square root of their variances (R = 1 indicates an exact agreement between signal). The PCC was used to assess the coordination between pairs of muscles. The PCC comparing all muscles was calculated for epochs of 20 data points progressively throughout the movements in order to assess dynamic muscular coordination between pairs of muscles of muscle groups.

## Results

### Activation

**Fig 2:**
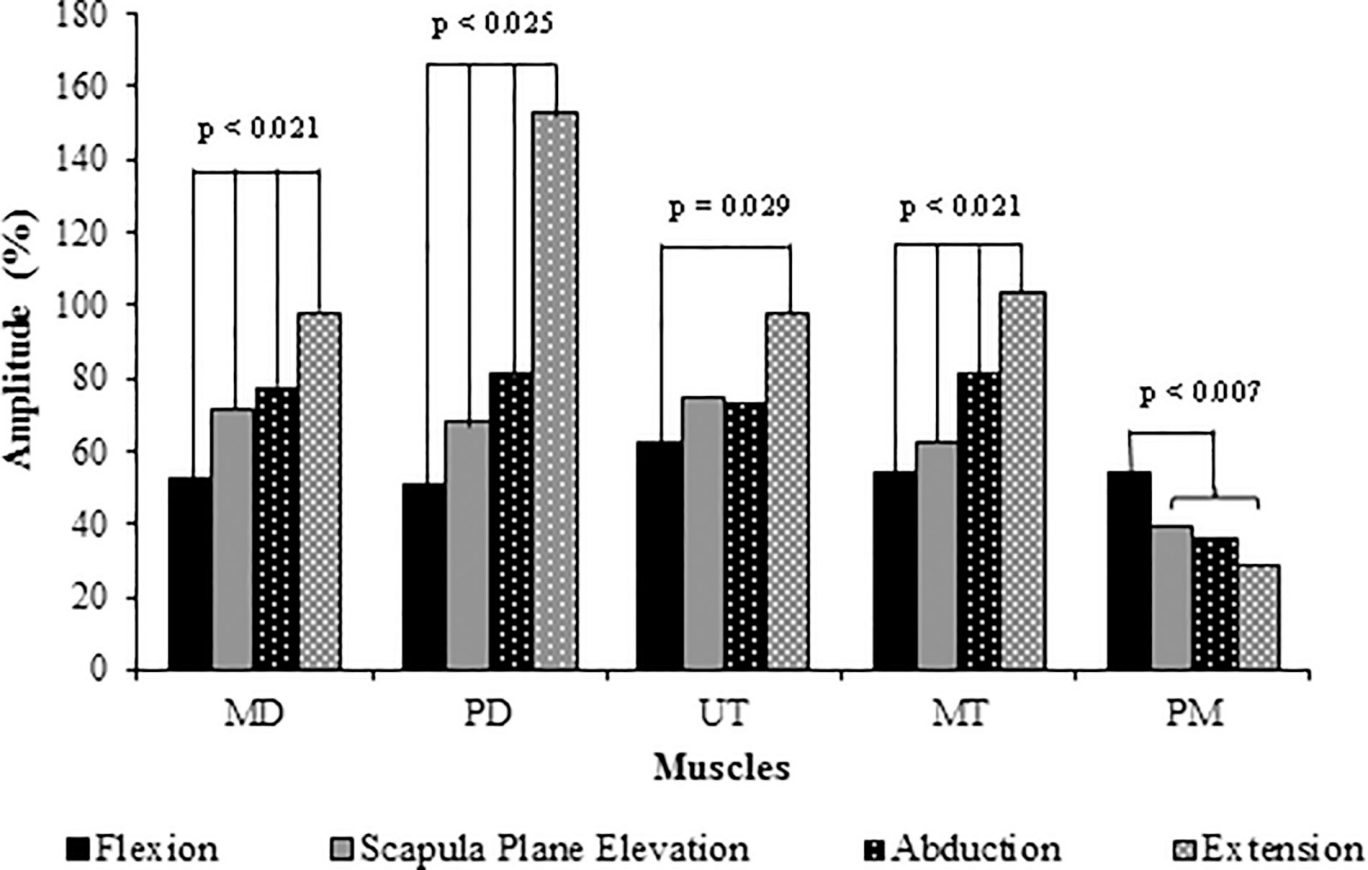
Mean signal amplitude during shoulder elevation across the different planes of shoulder movement. P values are given for significant comparisons.

**Fig 3:**
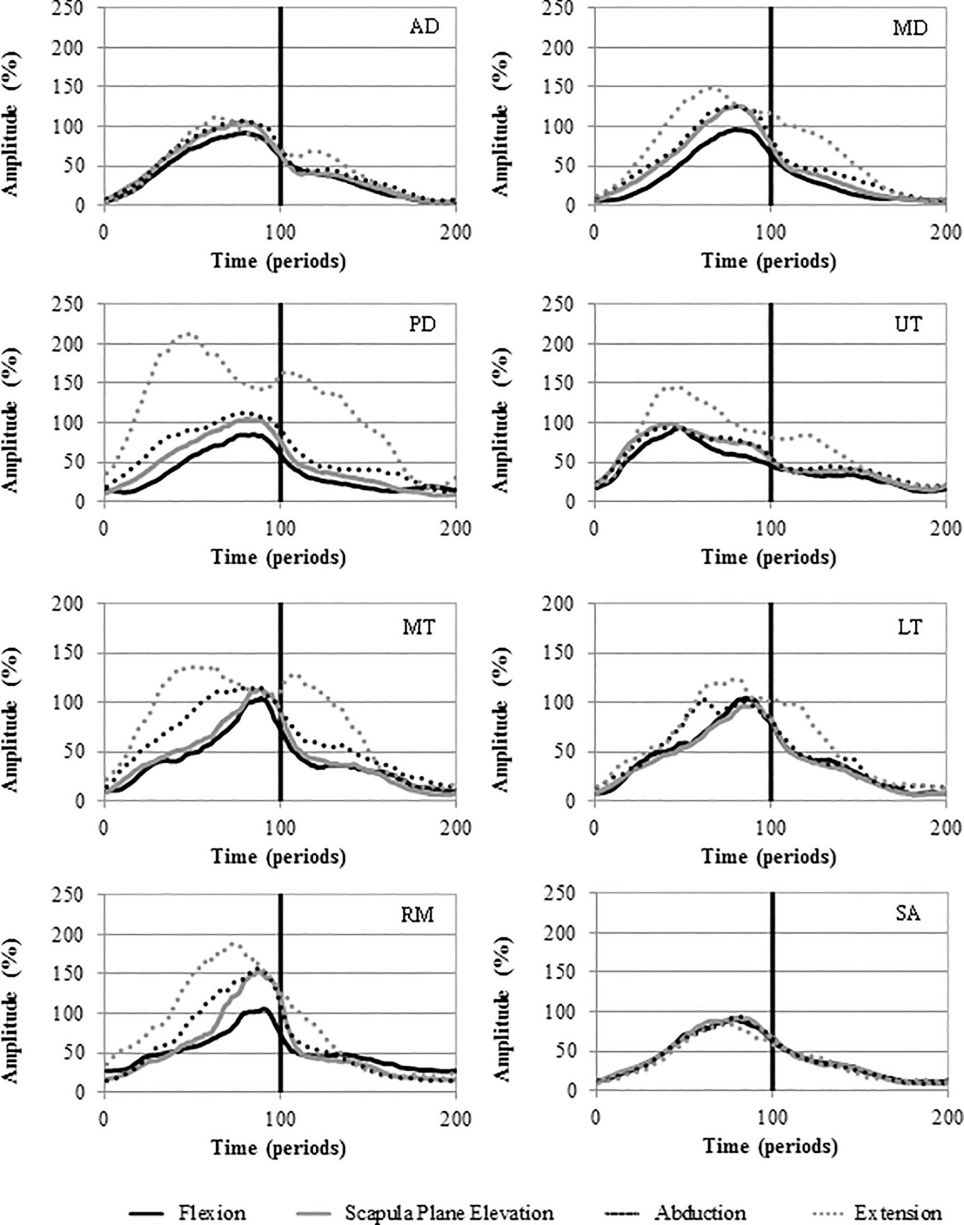
Ensemble average activation curves for the AD, MD, PD, UT, MT, LT, RM and SA. Mean EMG signal amplitude is presented as a function of time (periods). Elevation 0–100; Depression 100–200.

**Fig 4:**
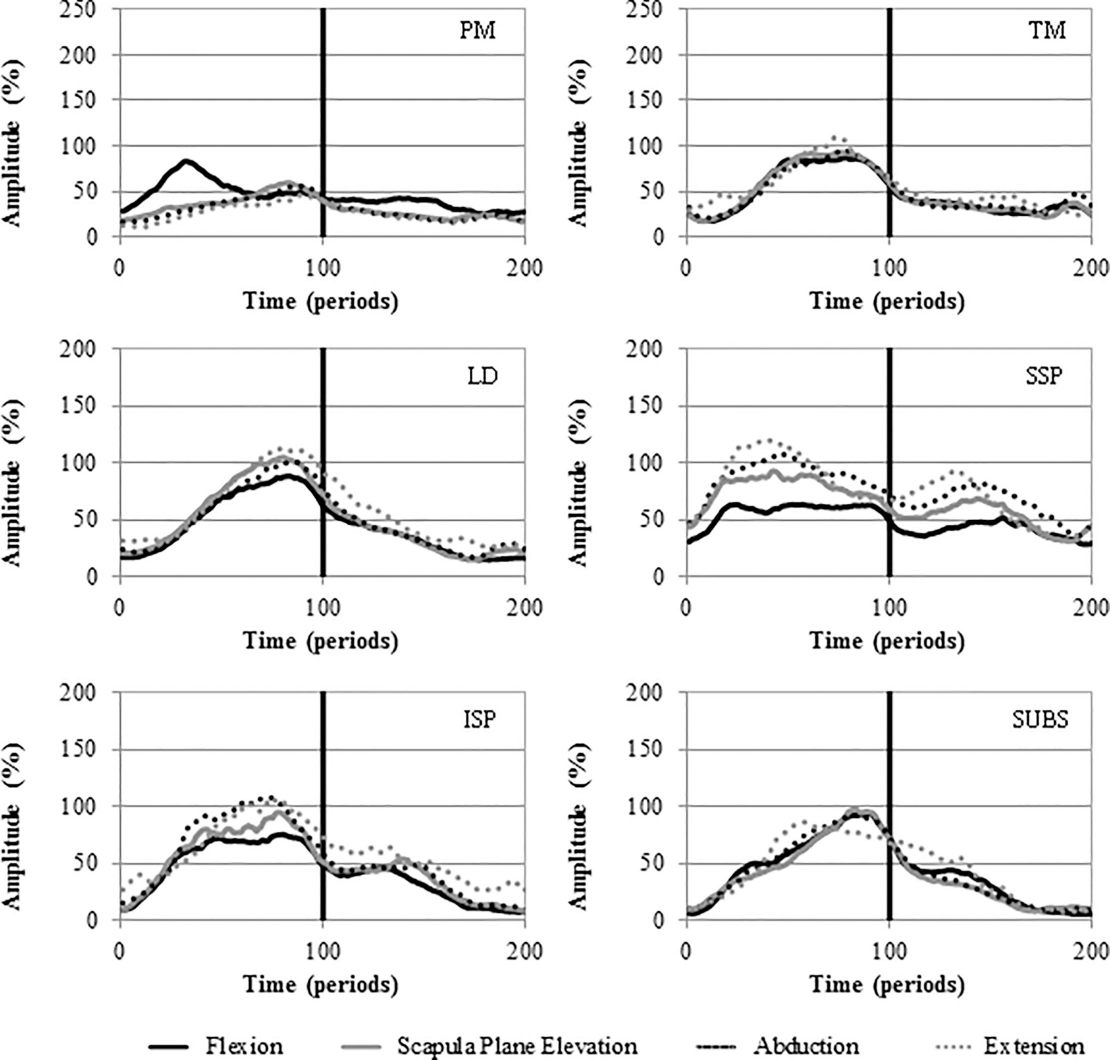
Ensemble average activation curves for the PM, TM, LD, SSP, ISP and SUBS. Mean EMG signal amplitude is presented as a function of time (periods). Elevation 0–100; Depression 100–200.

Table 1 gives the mean EMG amplitude for each muscle during the different planes of movement. Significantly higher activity was seen during arm elevation as compared to depression for all muscles studied except for RM during abduction and extension and SUBS during extension. Significant differences in signal amplitude were seen across all movements for MD, PD and MT. In addition, UT was significantly more active during extension than flexion and PM significantly more active during flexion as compared to the other movements (Fig 2). There were no significant differences in muscle activation between the movements for AD, LT, SA, TM, LD, SSP, ISP, SUBS, RM. Figs 3 and 4 depict the activation patterns for the individual muscles. The supplementary files reports the mean amplitude data disaggregated by gender (S1, S2, S3 and S4 Tables).

**Table 1:** Mean signal amplitude during shoulder elevation and depression in the different plane of shoulder motion.

Muscles	Flexion			Scapular Plane Elevation			Abduction			Extension		
	Elevation	Depression	*p* value^b^	Elevation	Depression	*p* value^b^	Elevation	Depression	*p* value^b^	Elevation	Depression	*p* value^b^
	Mean Amplitude (%)^a^	Mean Amplitude (%)^a^		Mean Amplitude (%)^a^	Mean Amplitude (%)^a^		Mean Amplitude (%)^a^	Mean Amplitude (%)^a^		Mean Amplitude (%)^a^	Mean Amplitude (%)^a^	
AD	59±2	24±2	**<0.001**	69±4	25±2	**<0.001**	71±5	30±3	**<0.001**	69±6	35±4	**<0.001**
MD	52±2	20±2	**<0.001**	72±5	24±2	**<0.001**	78±5	32±3	**<0.001**	98±8	52±4	**<0.001**
PD	51±2	22±2	**<0.001**	68±5	27±2	**<0.001**	81±7	38±4	**<0.001**	153±19	90±11	**0.001**
UT	62±2	28±2	**<0.001**	75±6	32±2	**<0.001**	74±5	35±2	**<0.001**	98±12	49±7	**<0.001**
MT	55±3	29±3	**<0.001**	63±4	29±2	**<0.001**	82±13	43±9	**<0.001**	104±10	61±8	**0.001**
LT	59±4	29±3	**<0.001**	57±4	27±3	**<0.001**	68±10	32±6	**<0.001**	78±12	44±6	**0.004**
RM	63±7	40±11	**0.002**	77±18	34±4	**0.046**	90±23	35±3	0.057	124±41	46±5	0.129
SA	58±2	27±2	**<0.001**	60±3	26±3	**<0.001**	57±3	26±3	**<0.001**	52±5	27±5	**<0.001**
TM	61±2	33±3	**<0.001**	64±4	33±3	**<0.001**	62±3	36±4	**<0.001**	69±4	39±4	**<0.001**
LD	58±2	31±3	**<0.001**	67±3	33±4	**<0.001**	63±4	35±4	**<0.001**	72±8	45±8	**<0.001**
PM	54±3	35±3	**<0.001**	39±5	24±3	**<0.001**	37±4	24±4	**<0.001**	29±3	23±3	**0.001**
SSP	57±4	41±5	**<0.001**	78±11	52±9	**<0.001**	88±14	64±11	**0.001**	90±19	61±14	**0.008**
ISP	57±3	28±3	**<0.001**	64±13	32±7	**0.005**	74±15	34±7	**0.013**	72±12	47±15	0.140
SUBS	58±2	28±2	**<0.001**	56±5	25±2	**<0.001**	59±5	26±3	**<0.001**	57±9	33±6	**0.010**

AD–anterior deltoid; MD–middle deltoid, PD–posterior deltoid; UT–upper trapezius; MT–middle trapezius; LT–lower trapezius; RM–rhomboid major; SA–serratus anterior; TM–teres major; LD–latissimus dorsi; PM–pectoralis major; SSP–supraspinatus; ISP–infraspinatus; SUBS–subscapularis

^a^ Values are means ± SEM

^b^ paired t-test comparing amplitude during each phase

### Timing

**Fig 5:**
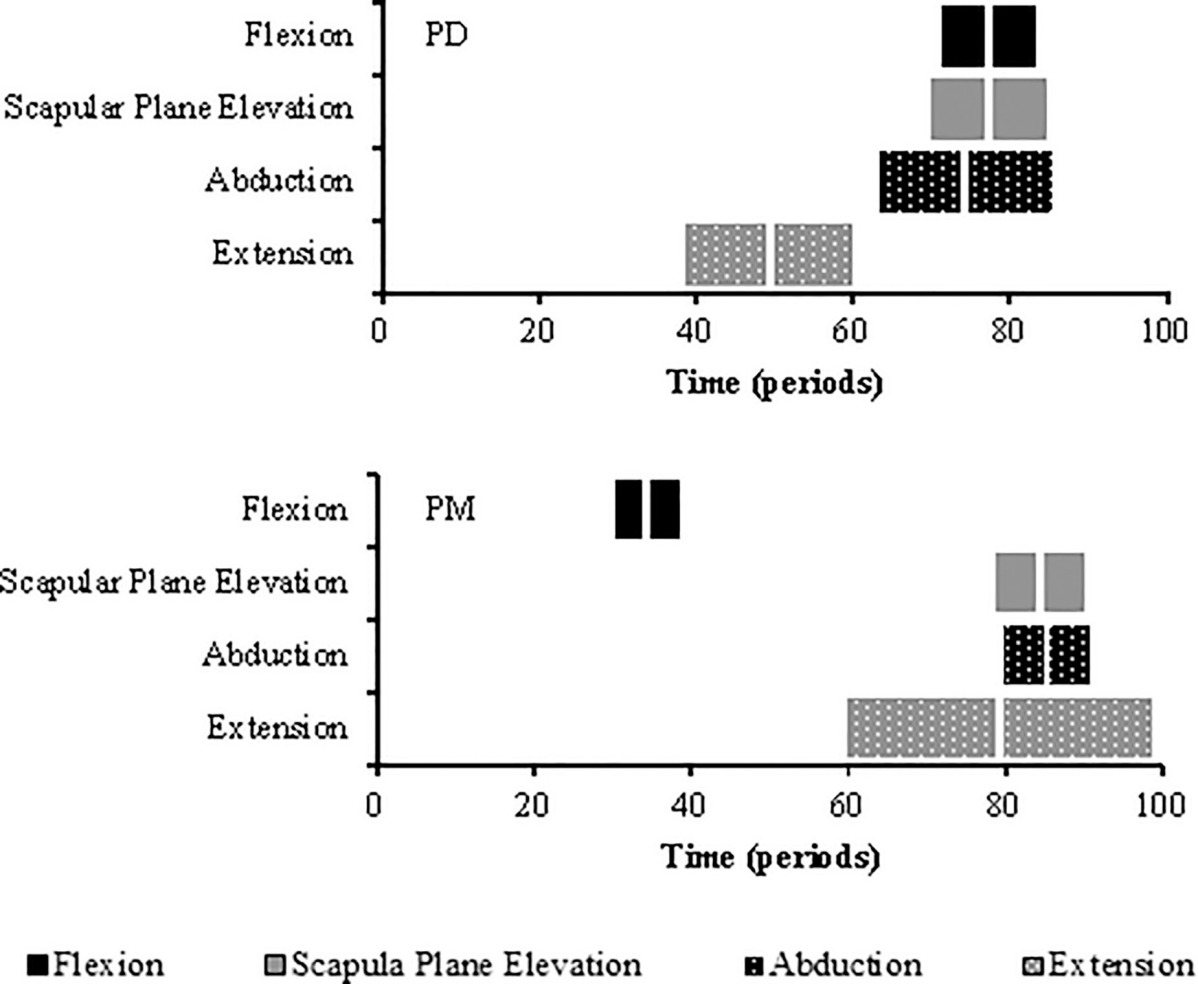
Box plots illustrating time of peak muscle amplitude for the PD and PM during shoulder elevation. Boxes show the interquartile range and the thick white line the median.

Peak activity of PD is seen significantly earlier in extension than in flexion (p<0.001), scapular plane elevation (p<0.001) and abduction (p = 0.001). Peak activity of PM is seen significantly earlier in flexion compared to scapular plane elevation (p = 0.004), abduction (p = 0.003) and extension (p = 0.011) (Fig 5). No muscles exhibited a double peak in their activation profile.

### Muscular coordination

**Fig 6:**
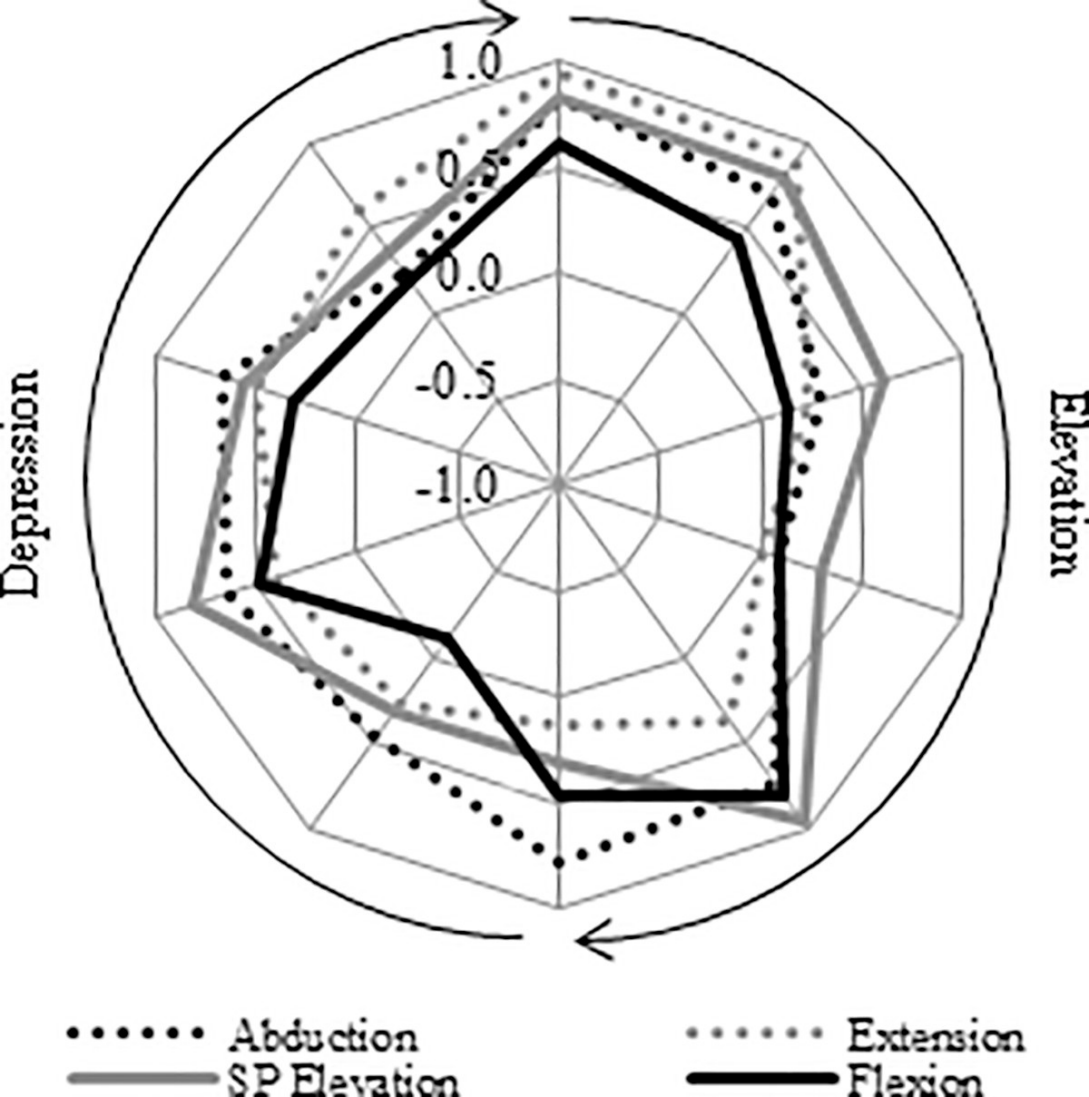
Dynamic coordination between the deltoid and rotator cuff muscle groups during the different planes of shoulder elevation as illustrated by the moving PCC.

Fig 6 illustrates the dynamic muscular coordination between the deltoid and rotator cuff muscle groups during shoulder elevation in the different planes of movement. Considering all movement planes together, coordination between the muscle groups was significantly higher during the initial (PCC = 0.79) and final (PCC = 0.74) stage of shoulder elevation compared to the mid-range (PCC = 0.34) (p = 0.020–0.035).

**Fig 7:**
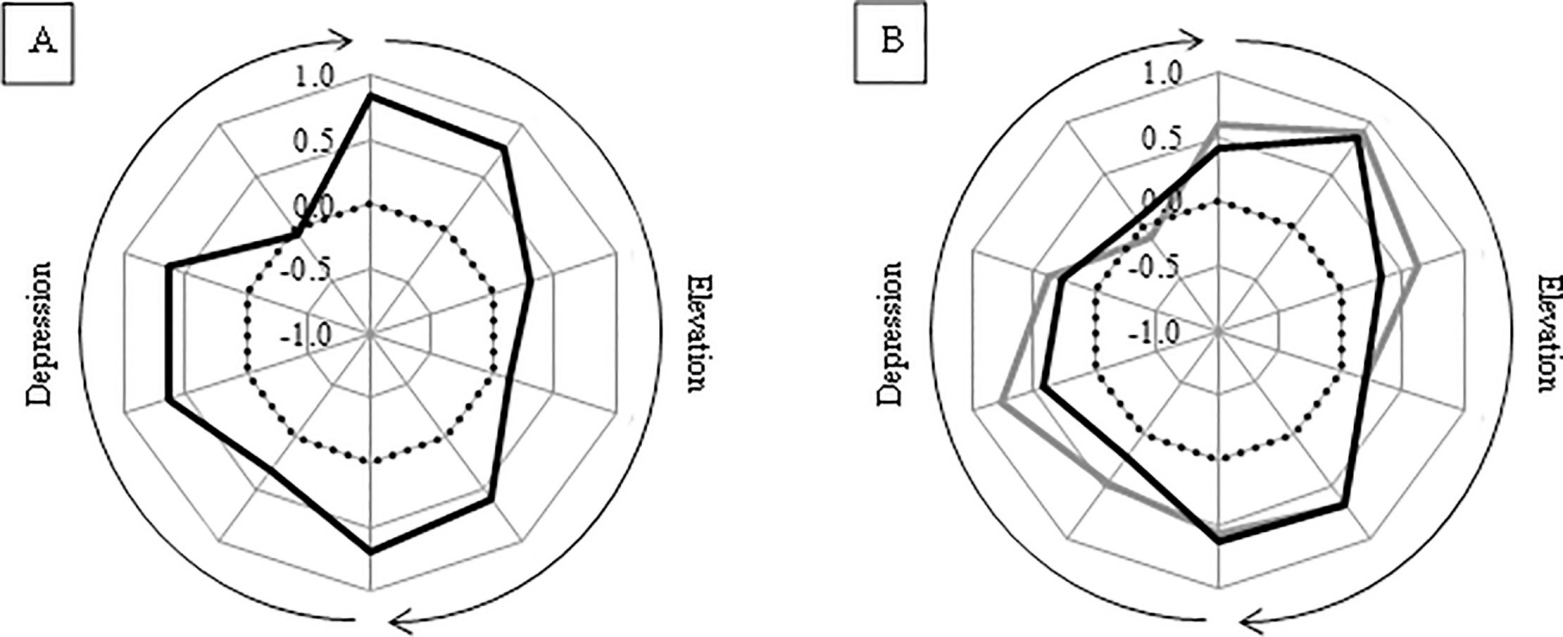
A—Moving PCC indicating the dynamic coordination between the UT/SA and MT/LT muscle groups. B—Moving PCC indicating the dynamic coordination between the TM and deltoid (black line) and LD and deltoid (grey line) muscle groups.

High coordination was seen between the UT/SA and the MT/LT muscle groups during the initial phases of shoulder elevation (PCC = 0.75–0.84). Considering the LD/TM as a functional group, the coordination with the deltoid is particularly high (PCC = 0.89) during the second stage of elevation (Fig 7). The coordination for all individual muscles comparisons for the different movement planes is presented within the supplementary data files (S5, S6, S7 and S8 Tables).

## Discussion

The shoulder is a complex system which relies on coordinated muscle activity to maintain stability [5–7]. Aberrant shoulder muscle coordination is considered to be an important aetiological component of shoulder impingement syndrome and shoulder instability [11, 12]. A recent randomised control trial has questioned the role of surgery in subacromial impingement syndrome and highlighted the important of rehabilitation [35]. However, an intimate knowledge of normal shoulder inter-muscular relationships is required if the pathological movement patterns of patients are going to be treated effectively with physiotherapy.

The results of the study are discussed below, firstly with respect to those muscles that act on the glenohumeral joint followed by those that comprise the scapulothoracic articulation. Broadly, muscles whose line of action is on the same side as the axis of movement are considered ‘movers’ and those on the opposite side, ‘stabilisers’.

### Glenohumeral muscles

Superior humeral head translation on the glenoid fossa, by the order of a few millimetres, during the initial stage of shoulder elevation has been well documented [3, 36, 37]. It occurs due to the lack of osseous congruity between the articular surfaces. Poppen and Walker, examining the resultant force vector during shoulder elevation, proposed that the superior subluxing force exerted by deltoid peaks at 60^o^ of abduction before decreasing thereafter [14]. Other muscles are required to have a stabilising effect to balance this superior subluxation and prevent the humeral head from impinging on the acromion.

The anatomical arrangement of the rotator cuff muscles is such that contraction exerts a compressive force between the humeral head and glenoid fossa which ‘stiffens’ the joint [8]. The dynamic inter-muscular coordination between the deltoid and rotator cuff muscle groups, as indicated by the moving PCC, showed a high correlation during the initial (PCC = 0.79) stage of shoulder elevation (Fig 6). This synchronous activity pattern shows that the muscles are working in a coordinated fashion with rotator cuff activation balancing the superior subluxing force of the deltoid. Additionally, the coordination between the deltoid and LD/TM groups is particularly high (PCC = 0.89) during the second stage of elevation (Fig 7B). The LD and TM both exert an adducting force that can balance the superior destabilising force of the deltoid, in a similar fashion to the rotator cuff [38]. Consequently, this study suggests a stabilising role for the rotator cuff, LD and TM during the initial phase of shoulder elevation.

In the mid-range of elevation the resultant force vector from the deltoid moves from exerting a superior subluxing force to pass through the glenoid in a more collinear fashion. At this time, the deltoid is generating its own compressive force across the GH joint which, as a consequence, is self-stabilising. Therefore, the requirement for other muscles to contribute to the stability during this phase is less. This suggestion is supported by the moving PCC results. Deltoid-rotator cuff coordination is significantly lower (PCC = 0.34) during the mid-range of elevation than at other times, and the moving PCC between the deltoid and LD/TM muscle group is also low, as illustrated in Fig 7B.

**Fig 8:**
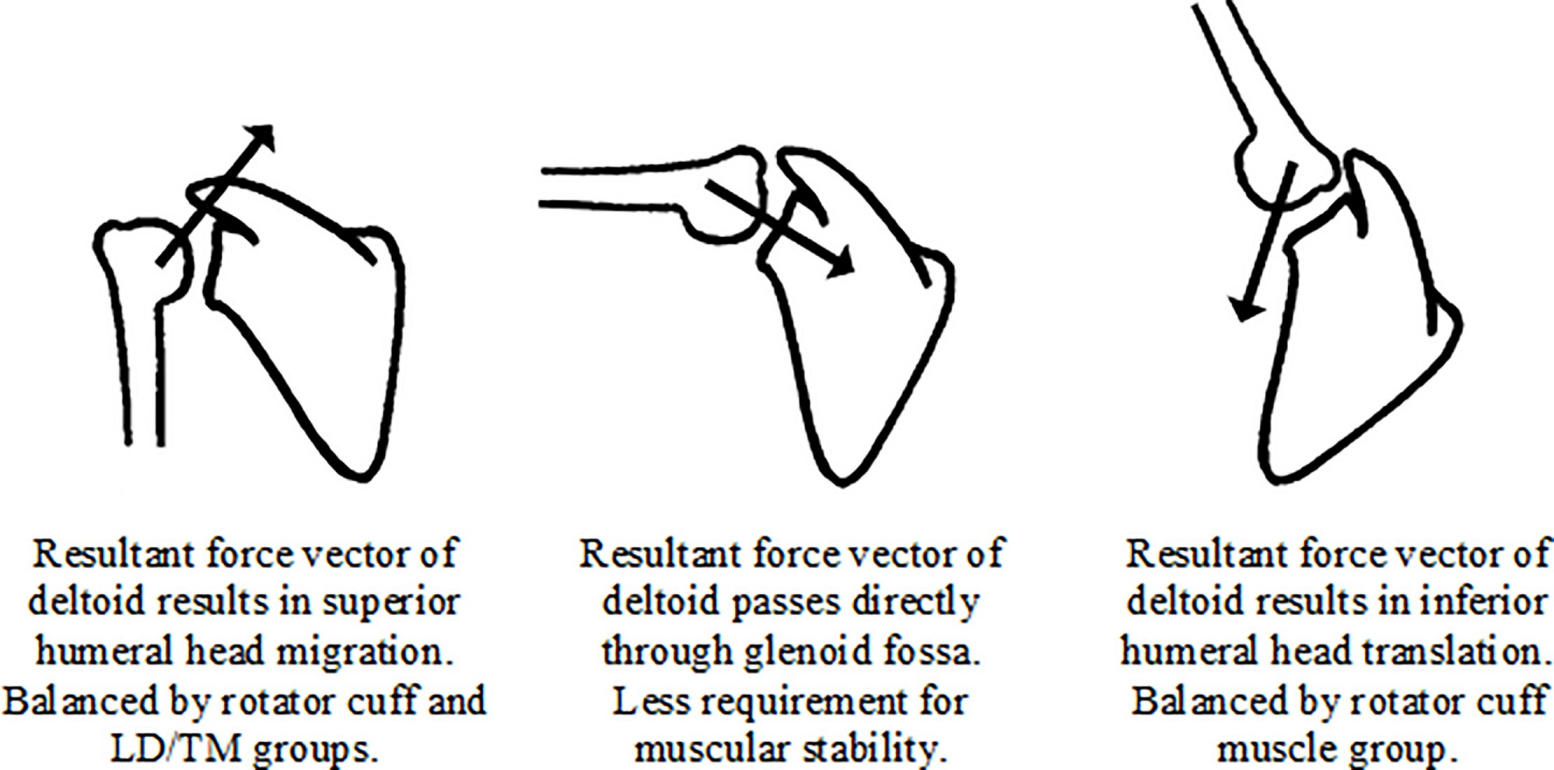
Schematic representation of muscular coordination at the GH joint during elevation. The arrow within each figure indicates gives a representation of the resultant force vector.

As shoulder elevation continues to progress, the humeral head translates inferiorly on the glenoid fossa [3, 36, 37], as the force vector exerted by the deltoid comes to pass below the axis of the glenoid. Accordingly, the requirement for other muscle groups to contribute to stability again increases. This is reflected by the high coordination between the deltoid and rotator cuff muscle groups at the end of elevation (PCC = 0.74). Coordination between the LD/TM and deltoid muscle groups is understandably low as (because of their line of action) synchronised activation would perpetuate inferior humeral head migration. Fig 8 is a schematic representation of the proposed coordination between muscle groups at the GH joint during shoulder elevation.

Considering the level of muscle activation, the PD was significantly more active during extension compared to movement in all other planes; this is consistent with its accepted function and was expected given its anatomical orientation and line of action [16]. Interestingly, the activity of the MD increased as the plane of shoulder elevation moved from flexion to extension. Similar findings were described in a recent study by Reed, although extension was not considered; in Reed’s study, MD activity was highest in the coronal plane, then decreased in the scapula plane before being lowest during elevation in a plane 30^o^ anterior to the scapula plane [19]. In this work, the activity of PM was significantly higher during flexion compared to movement in the other planes, and its peak activity occurred significantly earlier during flexion compared to the other movements. This again was anticipated given its primary accepted role as a shoulder flexor [39]. There were no significant differences in the level of muscle activation between the different movement planes for the AD, TM, LD, SSP, ISP or SUBS; this is in line with previous work [19].

### Scapulothoracic muscles

Scapula motion positions the glenoid effectively in space creating a basis for arm movement. The UT and SA elevate and upwardly rotate the scapula whilst the MT and LT counter this by depressing and downwardly rotating it. There is a high coordination between these functional muscle groups during the initial phase of shoulder elevation (Fig 7 (A)). This is proposed to reflect the establishment of a force couple which enables controlled scapula motion and the early glenoid positioning.

UT activity was significantly higher during extension compared to flexion. The UT inserts on the lateral third of the clavicle and contraction results in clavicular elevation. The clavicle retracts, elevates and rotates posteriorly on its axis during shoulder elevation. A kinematic study found greater clavicular elevation and retraction during shoulder elevation in the coronal as compared to the sagittal plane [40]. Increasing UT muscle activity as the plane of elevation moves posteriorly from flexion was therefore expected given the known kinematic patterns of the clavicle.

MT activity was greatest in extension and decreased as the plane of shoulder elevation moved anteriorly. This is consistent with the results of other studies [19, 41]. The scapula, during shoulder elevation, upwardly and internally rotates and tilts posteriorly. The scapulohumeral rhythm is known to decrease as the plane of shoulder elevation moves in an anterior arc from abduction to flexion. Thus the scapula remains in a relatively more adducted position during coronal as compared to sagittal plane elevation [42]. The MT attaches to the spine of the scapula and is responsible for scapula adduction. The MT activity seen in this study therefore reflects known kinematic patterns of the scapula. There were no significant differences in the activation of LT, SA, or RM between the movement planes.

### Limitations of this study

The potential for cross talk is a limitation of all EMG studies that employ surface electrodes. This is particularly pertinent to the shoulder due to its compact anatomy. Further, during dynamic movement there is also the potential for surface electrodes to move in relation to the underlying muscle belly. Nevertheless EMG has extensively been used within the literature to record muscle activation during dynamic shoulder movements. In this study the careful placement of appropriately sized electrodes according to accepted anatomical criteria limited the impact of any potential cross talk. Normalisation was performed in the time domain to correct for any inter-individual variation in the speed at which the movement protocol was undertaken. This could be improved by the use of concurrent motion capture, which would allow normalisation to GH elevation angle. Furthermore, normalisation to the mean EMG amplitude precludes any conclusions about absolute muscle activity levels. However, the primary aim of this work was to investigate inter-muscular coordination, for which normalisation in the time domain and to the mean amplitude is appropriate. Including additional planes of shoulder elevation between abduction and extension would also provide a more complete picture in this arc of movement. Finally, the conclusions drawn here remain an oversimplification. Muscles are not homogeneous, but rather have distinct segments that can act as different functional units [43]. However, evaluating coordination between all of these distinct segments would be unrealistically complicated at this stage.

## Conclusion

The coordination between muscle groups is dynamic and changes during a task as the requirements for stability alter. In broad outline, the destabilising force of the deltoid, during the initial stage of shoulder elevation, is balanced by the coordinated activity of the rotator cuff, LD and TM. Stability requirements are lower during the mid-range of elevation and this is reflected in the reduced coordination between these muscle groups. At the end-range of movement the demand for muscular stability again increases and here this is provided by the rotator cuff. It is suggested that this fuller analysis of shoulder function in health will improve understanding of the alterations associated with pathology, and assist development of more targeted, evidenced-based, treatment strategies.

## Supporting information

S1 Table Mean EMG amplitude during flexion. Mean amplitude data for flexion disaggregated by sex
(DOCX)

S2 Table Mean EMG amplitude during scapula plane elevation. Mean amplitude data for scapula plane elevation disaggregated by sex
(DOCX)

S3 Table Mean EMG amplitude during abduction. Mean amplitude data for abduction disaggregated by sex
(DOCX)

S4 Table Mean EMG amplitude during extension. Mean amplitude data for extension disaggregated by sex
(DOCX)

S5 Table Individual muscle flexion PCC. PCC comparing EMG between individual muscles during flexion.
(DOCX)

S6 Table Individual muscle scapula plane elevation PCC. PCC comparing EMG between individual muscles during scapula plane elevation.
(DOCX)

S7 Table Individual muscle abduction PCC. PCC comparing EMG between individual muscles during abduction.
(DOCX)

S8 Table Individual muscle extension PCC. PCC comparing EMG between individual muscles during extension.
(DOCX)

S1 Data Processed EMG data for the individual subjects during the different planes of movement. (XLSX)
