# Supplementary material for: Patterns of muscle coordination during dynamic glenohumeral joint elevation: An EMG study
Source: PLoS One. 2019 Feb 8;14(2):e0211800. doi: 10.1371/journal.pone.0211800 (PMC6368381; doi:10.1371/journal.pone.0211800)
Supplement: S1 Table — Mean amplitude data for flexion disaggregated by sex (DOCX) [file pone.0211800.s001.docx]

**S1 Table. Mean EMG Amplitude during Flexion.** Mean amplitude data for flexion disaggregated by sex

| Muscles | Flexion | | | | |
| --- | --- | --- | --- | --- | --- |
|  | Elevation | |  | Depression | |
|  | Males | Females |  | Males | Females |
|  | Mean Amplitude (%)^a^ | Mean Amplitude (%)^a^ |  | Mean Amplitude (%)^a^ | Mean Amplitude (%)^a^ |
| AD | 59±4 | 60±3 |  | 22±3 | 25±2 |
| MD | 48±3 | 57±3 |  | 16±2 | 23±2 |
| PD | 50±3 | 52±4 |  | 21±2 | 25±3 |
| UT | 59±4 | 66±2 |  | 26±2 | 30±3 |
| MT | 49±3 | 60±3 |  | 25±7 | 33±3 |
| LT | 56±8 | 62±4 |  | 21±2 | 35±5 |
| RM | 54±6 | 75±12 |  | 26±8 | 58±20 |
| SA | 58±3 | 58±2 |  | 25±4 | 29±2 |
| TM | 63±3 | 59±4 |  | 36±5 | 31±4 |
| LD | 54±3 | 62±4 |  | 25±2 | 37±5 |
| PM | 54±3 | 55±5 |  | 35±3 | 36±5 |
| SSP | 54±5 | 62±7 |  | 36±7 | 47±6 |
| ISP | 58±3 | 56±6 |  | 33±4 | 22±4 |
| SUBS | 54±3 | 61±3 |  | 25±4 | 31±2 |

AD – anterior deltoid; MD – middle deltoid, PD – posterior deltoid; UT – upper trapezius; MT – middle trapezius; LT – lower trapezius; RM – rhomboid major; SA – serratus anterior; TM – teres major; LD – latissimus dorsi; PM – pectoralis major; SSP – supraspinatus; ISP – infraspinatus; SUBS – subscapularis

^a^ Values are means ± SEM
