# Supplementary material for: Patterns of muscle coordination during dynamic glenohumeral joint elevation: An EMG study
Source: PLoS One. 2019 Feb 8;14(2):e0211800. doi: 10.1371/journal.pone.0211800 (PMC6368381; doi:10.1371/journal.pone.0211800)
Supplement: S2 Table — Mean amplitude data for scapula plane elevation disaggregated by sex (DOCX) [file pone.0211800.s002.docx]

**S2 Table. Mean EMG Amplitude during Scapula Plane Elevation.** Mean amplitude data for scapula plane elevation disaggregated by sex

| Muscles | Scapula Plane Elevation | | | | |
| --- | --- | --- | --- | --- | --- |
|  | Elevation | |  | Depression | |
|  | Males | Females |  | Males | Females |
|  | Mean Amplitude (%)^a^ | Mean Amplitude (%)^a^ |  | Mean Amplitude (%)^a^ | Mean Amplitude (%)^a^ |
| AD | 67±8 | 71±4 |  | 24±4 | 26±2 |
| MD | 70±9 | 74±5 |  | 23±4 | 25±2 |
| PD | 69±9 | 66±6 |  | 28±4 | 26±2 |
| UT | 70±9 | 79±8 |  | 30±2 | 34±4 |
| MT | 66±11 | 60±3 |  | 25±5 | 32±2 |
| LT | 51±10 | 61±3 |  | 20±3 | 31±4 |
| RM | 87±26 | 56±1 |  | 34±6 | 33±2 |
| SA | 59±6 | 61±2 |  | 24±5 | 28±3 |
| TM | 64±4 | 63±7 |  | 36±4 | 30±5 |
| LD | 68±6 | 66±4 |  | 27±3 | 39±6 |
| PM | 34±7 | 46±7 |  | 19±4 | 30±5 |
| SSP | 70±17 | 87±14 |  | 50±16 | 54±10 |
| ISP | 89±12 | 52±15 |  | 45±4 | 26±8 |
| SUBS | 54±7 | 59±7 |  | 20±2 | 29±3 |

AD – anterior deltoid; MD – middle deltoid, PD – posterior deltoid; UT – upper trapezius; MT – middle trapezius; LT – lower trapezius; RM – rhomboid major; SA – serratus anterior; TM – teres major; LD – latissimus dorsi; PM – pectoralis major; SSP – supraspinatus; ISP – infraspinatus; SUBS – subscapularis

^a^ Values are means ± SEM
