# Supplementary material for: Patterns of muscle coordination during dynamic glenohumeral joint elevation: An EMG study
Source: PLoS One. 2019 Feb 8;14(2):e0211800. doi: 10.1371/journal.pone.0211800 (PMC6368381; doi:10.1371/journal.pone.0211800)
Supplement: S3 Table — Mean amplitude data for abduction disaggregated by sex (DOCX) [file pone.0211800.s003.docx]

**S3 Table. Mean EMG Amplitude during Abduction.** Mean amplitude data for abduction disaggregated by sex

| Muscles | Abduction | | | | |
| --- | --- | --- | --- | --- | --- |
|  | Elevation | |  | Depression | |
|  | Males | Females |  | Males | Females |
|  | Mean Amplitude (%)^a^ | Mean Amplitude (%)^a^ |  | Mean Amplitude (%)^a^ | Mean Amplitude (%)^a^ |
| AD | 72±9 | 71±3 |  | 28±4 | 31±4 |
| MD | 78±8 | 78±5 |  | 31±4 | 33±3 |
| PD | 86±12 | 77±8 |  | 38±6 | 39±5 |
| UT | 69±5 | 78±8 |  | 31±2 | 39±4 |
| MT | 103±25 | 64±4 |  | 52±20 | 35±2 |
| LT | 71±21 | 65±7 |  | 33±14 | 32±3 |
| RM | 102±34 | 66±14 |  | 37±4 | 32±1 |
| SA | 59±6 | 56±4 |  | 24±5 | 27±3 |
| TM | 66±4 | 57±5 |  | 41±5 | 30±5 |
| LD | 66±5 | 60±5 |  | 31±4 | 40±7 |
| PM | 36±7 | 37±6 |  | 20±5 | 27±5 |
| SSP | 78±17 | 100±23 |  | 56±16 | 73±17 |
| ISP | 104±37 | 59±9 |  | 46±3 | 27±8 |
| SUBS | 57±7 | 61±7 |  | 23±4 | 31±2 |

AD – anterior deltoid; MD – middle deltoid, PD – posterior deltoid; UT – upper trapezius; MT – middle trapezius; LT – lower trapezius; RM – rhomboid major; SA – serratus anterior; TM – teres major; LD – latissimus dorsi; PM – pectoralis major; SSP – supraspinatus; ISP – infraspinatus; SUBS – subscapularis

^a^ Values are means ± SEM
