# Supplementary material for: Patterns of muscle coordination during dynamic glenohumeral joint elevation: An EMG study
Source: PLoS One. 2019 Feb 8;14(2):e0211800. doi: 10.1371/journal.pone.0211800 (PMC6368381; doi:10.1371/journal.pone.0211800)
Supplement: S4 Table — Mean amplitude data for extension disaggregated by sex (DOCX) [file pone.0211800.s004.docx]

**S4 Table. Mean EMG Amplitude during Extension.** Mean amplitude data for extension disaggregated by sex

| Muscles | Extension | | | | |
| --- | --- | --- | --- | --- | --- |
|  | Elevation | |  | Depression | |
|  | Males | Females |  | Males | Females |
|  | Mean Amplitude (%)^a^ | Mean Amplitude (%)^a^ |  | Mean Amplitude (%)^a^ | Mean Amplitude (%)^a^ |
| AD | 62±8 | 75±7 |  | 32±5 | 39±6 |
| MD | 91±10 | 105±11 |  | 49±6 | 54±6 |
| PD | 160±31 | 146±22 |  | 92±14 | 89±18 |
| UT | 75±9 | 120±19 |  | 38±6 | 59±11 |
| MT | 107±22 | 102±10 |  | 55±11 | 65±12 |
| LT | 63±18 | 91±16 |  | 39±12 | 48±6 |
| RM | 140±88 | 107±42 |  | 52±10 | 41±2 |
| SA | 46±6 | 55±6 |  | 24±8 | 28±6 |
| TM | 65±9 | 72±5 |  | 39±6 | 38±6 |
| LD | 64±10 | 79±14 |  | 33±5 | 57±15 |
| PM | 26±3 | 32±5 |  | 17±3 | 28±5 |
| SSP | 55±24 | 118±23 |  | 37±15 | 81±19 |
| ISP | 91±11 | 58±18 |  | 76±28 | 26±8 |
| SUBS | 56±12 | 59±14 |  | 35±11 | 31±4 |

AD – anterior deltoid; MD – middle deltoid, PD – posterior deltoid; UT – upper trapezius; MT – middle trapezius; LT – lower trapezius; RM – rhomboid major; SA – serratus anterior; TM – teres major; LD – latissimus dorsi; PM – pectoralis major; SSP – supraspinatus; ISP – infraspinatus; SUBS – subscapularis

^a^ Values are means ± SEM
