# Supplementary material for: Patterns of muscle coordination during dynamic glenohumeral joint elevation: An EMG study
Source: PLoS One. 2019 Feb 8;14(2):e0211800. doi: 10.1371/journal.pone.0211800 (PMC6368381; doi:10.1371/journal.pone.0211800)
Supplement: S5 Table — PCC comparing EMG between individual muscles during flexion. (DOCX) [file pone.0211800.s005.docx]

**S5 Table. Individual Muscle Flexion PCC.** PCC comparing EMG between individual muscles during flexion.

|  | AD | MD | PD | UT | MT | LT | SA | TM | LD | PM | SSP | ISP | SUBS | RM |
| --- | --- | --- | --- | --- | --- | --- | --- | --- | --- | --- | --- | --- | --- | --- |
| AD |  | 0.85 | 0.62 | 0.66 | 0.74 | 0.93 | 0.85 | 0.43 | 0.90 | 0.40 | 0.18 | 0.70 | 0.90 | 0.73 |
|  |  | 0.333 | **0.028** | **0.039** | 0.590 | 0.088 | 0.325 | **0.006** | **0.019** | **0.005** | **0.008** | 0.292 | 0.121 | 0.517 |
| MD | 0.87 |  | 0.79 | 0.51 | 0.72 | 0.88 | 0.76 | 0.38 | 0.83 | 0.25 | 0.04 | 0.50 | 0.82 | 0.66 |
|  | 0.333 |  | **0.046** | 0.053 | 0.145 | 0.620 | 0.376 | **0.003** | 0.302 | **0.002** | **0.005** | 0.381 | 0.771 | 0.587 |
| PD | 0.84 | 0.91 |  | 0.31 | 0.66 | 0.74 | 0.58 | 0.27 | 0.60 | 0.18 | -0.04 | 0.25 | 0.72 | 0.64 |
|  | **0.028** | **0.046** |  | 0.666 | 0.229 | 0.141 | 0.092 | **0.003** | 0.051 | 0.124 | **0.006** | **0.049** | 0.411 | 0.914 |
| UT | 0.46 | 0.31 | 0.37 |  | 0.53 | 0.65 | 0.59 | 0.34 | 0.72 | 0.44 | 0.47 | 0.66 | 0.62 | 0.51 |
|  | **0.039** | 0.053 | 0.666 |  | **0.046** | **0.007** | **0.010** | 0.662 | **0.006** | 0.878 | 0.438 | 0.433 | 0.106 | 0.016 |
| MT | 0.81 | 0.89 | 0.86 | 0.26 |  | 0.80 | 0.75 | 0.25 | 0.69 | 0.26 | 0.29 | 0.41 | 0.93 | 0.85 |
|  | 0.590 | 0.145 | 0.229 | **0.046** |  | 0.248 | 0.149 | **0.021** | 0.149 | 0.557 | 0.257 | 0.252 | 0.938 | 0.360 |
| LT | 0.87 | 0.89 | 0.87 | 0.32 | 0.92 |  | 0.86 | 0.44 | 0.84 | 0.24 | 0.20 | 0.62 | 0.93 | 0.78 |
|  | 0.088 | 0.620 | 0.141 | **0.007** | 0.248 |  | 0.466 | **0.025** | 0.319 | 0.817 | **0.038** | 0.303 | 0.148 | 0.748 |
| SA | 0.81 | 0.84 | 0.78 | 0.36 | 0.81 | 0.84 |  | 0.41 | 0.87 | 0.48 | 0.08 | 0.65 | 0.88 | 0.73 |
|  | 0.325 | 0.376 | 0.092 | **0.010** | 0.149 | 0.466 |  | **0.004** | 0.417 | **0.034** | **0.033** | 0.884 | 0.145 | 0.743 |
| TM | 0.80 | 0.76 | 0.73 | 0.41 | 0.61 | 0.72 | 0.77 |  | 0.44 | 0.21 | 0.06 | 0.61 | 0.35 | 0.21 |
|  | **0.006** | **0.003** | **0.003** | 0.662 | **0.021** | **0.025** | **0.004** |  | **0.006** | 0.367 | **0.010** | 0.622 | **0.036** | 0.212 |
| LD | 0.85 | 0.86 | 0.83 | 0.44 | 0.86 | 0.89 | 0.90 | 0.78 |  | 0.41 | 0.21 | 0.78 | 0.85 | 0.73 |
|  | **0.019** | 0.302 | 0.051 | **0.006** | 0.149 | 0.319 | 0.417 | **0.006** |  | **0.015** | 0.169 | 0.585 | 0.499 | 0.907 |
| PM | 0.09 | -0.07 | -0.03 | 0.43 | 0.15 | 0.20 | 0.16 | 0.05 | 0.07 |  | 0.15 | 0.60 | 0.41 | 0.14 |
|  | **0.005** | **0.002** | 0.124 | 0.878 | 0.557 | 0.817 | **0.034** | 0.367 | **0.015** |  | 0.726 | 0.165 | 0.146 | 0.197 |
| SSP | 0.51 | 0.44 | 0.40 | 0.61 | 0.39 | 0.48 | 0.32 | 0.42 | 0.39 | 0.22 |  | 0.24 | 0.34 | 0.32 |
|  | **0.008** | **0.005** | **0.006** | 0.438 | 0.257 | **0.038** | **0.033** | **0.010** | 0.169 | 0.726 |  | 0.280 | 0.209 | 0.201 |
| ISP | 0.86 | 0.60 | 0.68 | 0.54 | 0.73 | 0.87 | 0.63 | 0.69 | 0.73 | 0.35 | 0.41 |  | 0.77 | 0.56 |
|  | 0.292 | 0.381 | **0.049** | 0.433 | 0.252 | 0.303 | 0.884 | 0.622 | 0.585 | 0.165 | 0.280 |  | 0.548 | 0.617 |
| SUBS | 0.83 | 0.84 | 0.80 | 0.38 | 0.93 | 0.90 | 0.79 | 0.65 | 0.82 | 0.17 | 0.40 | 0.84 |  | 0.83 |
|  | 0.121 | 0.771 | 0.411 | 0.106 | 0.938 | 0.148 | 0.145 | **0.036** | 0.499 | 0.146 | 0.209 | 0.548 |  | 0.306 |
| RM | 0.65 | 0.71 | 0.65 | 0.21 | 0.77 | 0.75 | 0.70 | 0.53 | 0.72 | 0.39 | 0.46 | 0.84 | 0.75 |  |
|  | 0.517 | 0.587 | 0.914 | 0.016 | 0.360 | 0.748 | 0.743 | 0.212 | 0.907 | 0.197 | 0.201 | 0.617 | 0.306 |  |

Grey half (bottom left) gives muscle coordination for arm elevation and the white half (top right) for arm depression. PCC – Pearson correlation coefficient. The p-values given report a paired samples t-test comparing phases; significant comparisons (p<0.050) in bold;
