# Supplementary material for: Patterns of muscle coordination during dynamic glenohumeral joint elevation: An EMG study
Source: PLoS One. 2019 Feb 8;14(2):e0211800. doi: 10.1371/journal.pone.0211800 (PMC6368381; doi:10.1371/journal.pone.0211800)
Supplement: S6 Table — PCC comparing EMG between individual muscles during scapula plane elevation. (DOCX) [file pone.0211800.s006.docx]

**S6 Table. Individual Muscle Scapula Plane Elevation PCC.** PCC comparing EMG between individual muscles during scapula plane elevation.

|  | AD | MD | PD | UT | MT | LT | SA | TM | LD | PM | SSP | ISP | SUBS | RM |
| --- | --- | --- | --- | --- | --- | --- | --- | --- | --- | --- | --- | --- | --- | --- |
| AD |  | 0.92 | 0.82 | 0.67 | 0.91 | 0.93 | 0.79 | 0.35 | 0.79 | 0.55 | 0.57 | 0.84 | 0.80 | 0.88 |
|  |  | 0.187 | 0.801 | **0.038** | **0.009** | **0.031** | 0.606 | **0.004** | 0.526 | 0.184 | 0.967 | 0.401 | 0.872 | 0.628 |
| MD | 0.90 |  | 0.89 | 0.62 | 0.91 | 0.95 | 0.82 | 0.43 | 0.82 | 0.57 | 0.46 | 0.77 | 0.84 | 0.88 |
|  | 0.187 |  | 0.967 | **0.016** | 0.297 | 0.101 | 0.646 | **0.004** | 0.172 | 0.251 | 0.810 | 0.341 | 0.968 | 0.093 |
| PD | 0.83 | 0.89 |  | 0.66 | 0.92 | 0.93 | 0.83 | 0.41 | 0.76 | 0.60 | 0.52 | 0.76 | 0.82 | 0.92 |
|  | 0.801 | 0.967 |  | 0.078 | 0.204 | 0.059 | 0.450 | **0.005** | 0.094 | 0.128 | 0.341 | 0.896 | 0.660 | 0.424 |
| UT | 0.45 | 0.36 | 0.47 |  | 0.68 | 0.65 | 0.59 | 0.34 | 0.62 | 0.41 | 0.66 | 0.88 | 0.55 | 0.57 |
|  | **0.038** | **0.016** | 0.078 |  | 0.121 | 0.106 | **0.011** | 0.674 | **0.020** | 0.645 | 0.214 | 0.128 | 0.088 | 0.365 |
| MT | 0.78 | 0.87 | 0.84 | 0.45 |  | 0.94 | 0.86 | 0.38 | 0.83 | 0.48 | 0.57 | 0.93 | 0.84 | 0.93 |
|  | **0.009** | 0.297 | 0.204 | 0.121 |  | 0.291 | 0.412 | 0.115 | 0.860 | 0.082 | 0.509 | 1.000 | 0.517 | 0.646 |
| LT | 0.84 | 0.92 | 0.89 | 0.36 | 0.88 |  | 0.90 | 0.46 | 0.80 | 0.46 | 0.57 | 0.72 | 0.80 | 0.91 |
|  | **0.031** | 0.101 | 0.059 | 0.106 | 0.291 |  | 0.102 | 0.070 | 0.379 | 0.109 | 0.648 | 1.000 | 0.413 | 0.403 |
| SA | 0.72 | 0.76 | 0.76 | 0.31 | 0.81 | 0.73 |  | 0.46 | 0.78 | 0.57 | 0.45 | 0.68 | 0.76 | 0.83 |
|  | 0.606 | 0.646 | 0.450 | **0.011** | 0.412 | 0.102 |  | 0.119 | 0.994 | 0.236 | 0.398 | 0.245 | 0.793 | 0.381 |
| TM | 0.81 | 0.85 | 0.79 | 0.41 | 0.67 | 0.76 | 0.71 |  | 0.48 | 0.41 | 0.30 | 0.54 | 0.34 | 0.37 |
|  | **0.004** | **0.004** | **0.005** | 0.674 | 0.115 | 0.070 | 0.119 |  | **0.012** | 0.167 | 0.162 | 0.430 | **0.049** | 0.115 |
| LD | 0.84 | 0.92 | 0.88 | 0.38 | 0.85 | 0.90 | 0.78 | 0.82 |  | 0.59 | 0.49 | 0.72 | 0.78 | 0.90 |
|  | 0.526 | 0.172 | 0.094 | **0.020** | 0.860 | 0.379 | 0.994 | **0.012** |  | 0.357 | 0.692 | 0.806 | 0.456 | 0.499 |
| PM | 0.70 | 0.70 | 0.79 | 0.48 | 0.82 | 0.73 | 0.75 | 0.56 | 0.71 |  | 0.09 | 0.86 | 0.50 | 0.45 |
|  | 0.184 | 0.251 | 0.128 | 0.645 | 0.082 | 0.109 | 0.236 | 0.167 | 0.357 |  | 0.216 | 0.336 | 0.060 | 0.293 |
| SSP | 0.57 | 0.43 | 0.43 | 0.78 | 0.45 | 0.47 | 0.35 | 0.50 | 0.45 | 0.40 |  | 0.83 | 0.42 | 0.68 |
|  | 0.967 | 0.810 | 0.341 | 0.214 | 0.509 | 0.648 | 0.398 | 0.162 | 0.692 | 0.216 |  | 0.241 | 0.614 | 0.888 |
| ISP | 0.79 | 0.67 | 0.75 | 0.60 | 0.69 | 0.63 | 0.53 | 0.69 | 0.71 | 0.82 | 0.71 |  | 0.82 | 0.91 |
|  | 0.401 | 0.341 | 0.896 | 0.128 | 1.000 | 1.000 | 0.245 | 0.430 | 0.806 | 0.336 | 0.241 |  | 0.808 | 1.000 |
| SUBS | 0.79 | 0.84 | 0.80 | 0.29 | 0.89 | 0.86 | 0.78 | 0.72 | 0.82 | 0.80 | 0.52 | 0.85 |  | 0.82 |
|  | 0.872 | 0.968 | 0.660 | 0.088 | 0.517 | 0.413 | 0.793 | **0.049** | 0.456 | 0.060 | 0.614 | 0.808 |  | 0.402 |
| RM | 0.84 | 0.94 | 0.84 | 0.37 | 0.91 | 0.94 | 0.76 | 0.77 | 0.92 | 0.69 | 0.71 | 0.63 | 0.90 |  |
|  | 0.628 | 0.093 | 0.424 | 0.365 | 0.646 | 0.403 | 0.381 | 0.115 | 0.499 | 0.293 | 0.888 | 1.000 | 0.402 |  |

Grey half (bottom left) gives muscle coordination for arm elevation and the white half (top right) for arm depression. PCC – Pearson correlation coefficient. The p-values given report a paired samples t-test comparing phases; significant comparisons (p<0.050) in bold;
