# Supplementary material for: Patterns of muscle coordination during dynamic glenohumeral joint elevation: An EMG study
Source: PLoS One. 2019 Feb 8;14(2):e0211800. doi: 10.1371/journal.pone.0211800 (PMC6368381; doi:10.1371/journal.pone.0211800)
Supplement: S7 Table — PCC comparing EMG between individual muscles during abduction. (DOCX) [file pone.0211800.s007.docx]

**S7 Table. Individual Muscle Abduction PCC.** PCC comparing EMG between individual muscles during abduction.

|  | AD | MD | PD | UT | MT | LT | SA | TM | LD | PM | SSP | ISP | SUBS | RM |
| --- | --- | --- | --- | --- | --- | --- | --- | --- | --- | --- | --- | --- | --- | --- |
| AD |  | 0.92 | 0.77 | 0.63 | 0.82 | 0.76 | 0.77 | 0.27 | 0.76 | 0.50 | 0.55 | 0.84 | 0.82 | 0.86 |
|  |  | 0.553 | 0.668 | 0.327 | 0.828 | 0.296 | 0.655 | **<0.001** | 0.318 | **0.028** | 0.935 | 0.609 | 0.662 | 0.583 |
| MD | 0.91 |  | 0.87 | 0.63 | 0.86 | 0.81 | 0.85 | 0.39 | 0.81 | 0.52 | 0.51 | 0.76 | 0.88 | 0.90 |
|  | 0.553 |  | 0.140 | **0.038** | 0.928 | 0.058 | 0.966 | **0.002** | 0.448 | 0.066 | 0.483 | 0.522 | 0.243 | 0.996 |
| PD | 0.79 | 0.79 |  | 0.61 | 0.76 | 0.72 | 0.79 | 0.38 | 0.72 | 0.52 | 0.43 | 0.79 | 0.80 | 0.77 |
|  | 0.668 | 0.140 |  | 0.521 | 0.700 | 0.426 | 0.342 | **0.011** | 0.880 | 0.178 | 0.897 | 0.904 | 0.588 | 0.378 |
| UT | 0.55 | 0.44 | 0.55 |  | 0.63 | 0.62 | 0.57 | 0.24 | 0.55 | 0.40 | 0.58 | 0.77 | 0.54 | 0.58 |
|  | 0.327 | **0.038** | 0.521 |  | 0.680 | 0.439 | 0.119 | 0.124 | 0.276 | 0.604 | 0.425 | 0.290 | 0.614 | 0.780 |
| MT | 0.84 | 0.86 | 0.80 | 0.58 |  | 0.92 | 0.90 | 0.19 | 0.69 | 0.48 | 0.50 | 0.86 | 0.95 | 0.96 |
|  | 0.828 | 0.928 | 0.700 | 0.680 |  | **0.047** | **0.030** | **0.038** | 0.335 | 0.131 | 0.777 | 1.000 | 0.271 | 0.738 |
| LT | 0.83 | 0.87 | 0.76 | 0.52 | 0.80 |  | 0.89 | 0.31 | 0.69 | 0.55 | 0.40 | 0.66 | 0.95 | 0.97 |
|  | 0.296 | 0.058 | 0.426 | 0.439 | **0.047** |  | 0.074 | **0.029** | 0.130 | 0.186 | 0.726 | 1.000 | 0.079 | 0.207 |
| SA | 0.80 | 0.84 | 0.72 | 0.43 | 0.76 | 0.85 |  | 0.35 | 0.79 | 0.47 | 0.40 | 0.67 | 0.93 | 0.93 |
|  | 0.655 | 0.966 | 0.342 | 0.119 | **0.030** | 0.074 |  | **0.005** | 0.612 | **0.046** | 0.961 | 0.183 | **0.042** | 0.272 |
| TM | 0.82 | 0.83 | 0.73 | 0.45 | 0.65 | 0.74 | 0.81 |  | 0.47 | 0.41 | 0.10 | 0.56 | 0.35 | 0.23 |
|  | **<0.001** | **0.002** | **0.011** | 0.124 | **0.038** | **0.029** | **0.005** |  | 0.065 | **0.033** | 0.079 | 0.214 | 0.066 | 0.169 |
| LD | 0.83 | 0.86 | 0.73 | 0.46 | 0.84 | 0.82 | 0.83 | 0.78 |  | 0.66 | 0.45 | 0.71 | 0.79 | 0.89 |
|  | 0.318 | 0.448 | 0.880 | 0.276 | 0.335 | 0.130 | 0.612 | 0.065 |  | 0.095 | 0.868 | 0.104 | 0.621 | 0.944 |
| PM | 0.80 | 0.77 | 0.70 | 0.47 | 0.81 | 0.74 | 0.74 | 0.70 | 0.81 |  | 0.11 | 0.54 | 0.57 | 0.63 |
|  | **0.028** | 0.066 | 0.178 | 0.604 | 0.131 | 0.186 | **0.046** | **0.033** | 0.095 |  | **0.043** | 0.133 | 0.264 | 0.304 |
| SSP | 0.54 | 0.41 | 0.44 | 0.68 | 0.44 | 0.32 | 0.41 | 0.47 | 0.43 | 0.40 |  | 0.85 | 0.46 | 0.79 |
|  | 0.935 | 0.483 | 0.897 | 0.425 | 0.777 | 0.726 | 0.961 | 0.079 | 0.868 | **0.043** |  | 0.094 | 0.838 | 0.831 |
| ISP | 0.80 | 0.68 | 0.80 | 0.63 | 0.87 | 0.72 | 0.61 | 0.74 | 0.76 | 0.78 | 0.76 |  | 0.63 | 0.77 |
|  | 0.609 | 0.522 | 0.904 | 0.290 | 1.000 | 1.000 | 0.183 | 0.214 | 0.104 | 0.133 | 0.094 |  | 0.137 | 1.000 |
| SUBS | 0.80 | 0.81 | 0.82 | 0.48 | 0.89 | 0.86 | 0.82 | 0.69 | 0.82 | 0.74 | 0.43 | 0.83 |  | 0.98 |
|  | 0.662 | 0.243 | 0.588 | 0.614 | 0.271 | 0.079 | **0.042** | 0.066 | 0.621 | 0.264 | 0.838 | 0.137 |  | 0.198 |
| RM | 0.88 | 0.90 | 0.82 | 0.64 | 0.97 | 0.88 | 0.84 | 0.75 | 0.90 | 0.90 | 0.74 | 0.84 | 0.89 |  |
|  | 0.583 | 0.996 | 0.378 | 0.780 | 0.738 | 0.207 | 0.272 | 0.169 | 0.944 | 0.304 | 0.831 | 1.000 | 0.198 |  |

Grey half (bottom left) gives muscle coordination for arm elevation and the white half (top right) for arm depression. PCC – Pearson correlation coefficient. The p-values given report a paired samples t-test comparing phases; significant comparisons (p<0.050) in bold;
