# Supplementary material for: Patterns of muscle coordination during dynamic glenohumeral joint elevation: An EMG study
Source: PLoS One. 2019 Feb 8;14(2):e0211800. doi: 10.1371/journal.pone.0211800 (PMC6368381; doi:10.1371/journal.pone.0211800)
Supplement: S8 Table — PCC comparing EMG between individual muscles during extension. (DOCX) [file pone.0211800.s008.docx]

**S8 Table. Individual Muscle Extension PCC.** PCC comparing EMG between individual muscles during extension.

|  | AD | MD | PD | UT | MT | LT | SA | TM | LD | PM | SSP | ISP | SUBS | RM |
| --- | --- | --- | --- | --- | --- | --- | --- | --- | --- | --- | --- | --- | --- | --- |
| AD |  | 0.89 | 0.80 | 0.79 | 0.86 | 0.82 | 0.81 | 0.38 | 0.74 | 0.34 | 0.78 | 0.75 | 0.75 | 0.77 |
|  |  | 0.276 | **<0.001** | 0.103 | 0.051 | 0.055 | 0.636 | 0.161 | 0.381 | **0.045** | **0.016** | 0.301 | 0.523 | 0.213 |
| MD | 0.84 |  | 0.91 | 0.80 | 0.87 | 0.80 | 0.84 | 0.53 | 0.78 | 0.34 | 0.77 | 0.75 | 0.80 | 0.85 |
|  | 0.276 |  | **0.002** | 0.118 | 0.074 | 0.155 | 0.240 | 0.549 | 0.493 | **0.045** | 0.144 | 0.237 | 0.732 | 0.264 |
| PD | 0.47 | 0.59 |  | 0.75 | 0.85 | 0.75 | 0.79 | 0.60 | 0.79 | 0.31 | 0.72 | 0.71 | 0.78 | 0.87 |
|  | **<0.001** | **0.002** |  | 0.549 | 0.061 | **0.002** | **0.002** | **0.047** | **0.001** | 0.426 | 0.631 | **0.038** | 0.107 | 0.421 |
| UT | 0.67 | 0.65 | 0.68 |  | 0.86 | 0.82 | 0.74 | 0.42 | 0.62 | 0.34 | 0.66 | 0.53 | 0.64 | 0.88 |
|  | 0.103 | 0.118 | 0.549 |  | 0.105 | **0.004** | **0.014** | 0.503 | **0.007** | 0.271 | 0.552 | 0.590 | 0.711 | 0.161 |
| MT | 0.68 | 0.77 | 0.71 | 0.75 |  | 0.94 | 0.85 | 0.40 | 0.77 | 0.41 | 0.66 | 0.72 | 0.84 | 0.98 |
|  | 0.051 | 0.074 | 0.061 | 0.105 |  | **0.021** | **0.002** | 0.889 | **0.037** | 0.667 | 0.551 | 1.000 | 0.511 | 0.230 |
| LT | 0.70 | 0.71 | 0.36 | 0.53 | 0.68 |  | 0.86 | 0.32 | 0.74 | 0.47 | 0.47 | 0.59 | 0.84 | 0.96 |
|  | 0.055 | 0.155 | **0.002** | **0.004** | **0.021** |  | **0.006** | 0.297 | 0.586 | 0.165 | 0.122 | 1.000 | 0.560 | 0.075 |
| SA | 0.77 | 0.78 | 0.35 | 0.48 | 0.61 | 0.72 |  | 0.45 | 0.79 | 0.38 | 0.65 | 0.64 | 0.78 | 0.90 |
|  | 0.636 | 0.240 | **0.002** | **0.014** | **0.002** | **0.006** |  | 0.182 | 0.585 | **0.007** | 0.087 | 0.734 | 0.697 | 0.047 |
| TM | 0.55 | 0.60 | 0.29 | 0.34 | 0.43 | 0.49 | 0.62 |  | 0.61 | 0.27 | 0.38 | 0.63 | 0.43 | 0.67 |
|  | 0.161 | 0.549 | **0.047** | 0.503 | 0.889 | 0.297 | 0.182 |  | 0.375 | 0.098 | 0.696 | 0.423 | 0.098 | 0.522 |
| LD | 0.68 | 0.73 | 0.31 | 0.37 | 0.46 | 0.78 | 0.81 | 0.69 |  | 0.41 | 0.49 | 0.72 | 0.80 | 0.77 |
|  | 0.381 | 0.493 | **0.001** | **0.007** | **0.037** | 0.586 | 0.585 | 0.375 |  | **0.045** | 0.136 | 0.971 | 0.888 | 0.123 |
| PM | 0.66 | 0.71 | 0.45 | 0.53 | 0.51 | 0.68 | 0.78 | 0.54 | 0.76 |  | -0.05 | 0.25 | 0.35 | 0.55 |
|  | **0.045** | **0.045** | 0.426 | 0.271 | 0.667 | 0.165 | **0.007** | 0.098 | **0.045** |  | 0.080 | 0.156 | 0.062 | 0.427 |
| SSP | 0.50 | 0.62 | 0.79 | 0.76 | 0.77 | 0.10 | 0.32 | 0.30 | 0.24 | 0.47 |  | 0.77 | 0.57 | 0.51 |
|  | **0.016** | 0.144 | 0.631 | 0.552 | 0.551 | 0.122 | 0.087 | 0.696 | 0.136 | 0.080 |  | 0.261 | 0.289 | 0.232 |
| ISP | 0.65 | 0.59 | 0.27 | 0.44 | 0.33 | 0.40 | 0.72 | 0.76 | 0.71 | 0.81 | 0.49 |  | 0.77 | 0.70 |
|  | 0.301 | 0.237 | **0.038** | 0.590 | 1.000 | 1.000 | 0.734 | 0.423 | 0.971 | 0.156 | 0.261 |  | 0.723 | 0.906 |
| SUBS | 0.71 | 0.78 | 0.59 | 0.59 | 0.73 | 0.92 | 0.82 | 0.65 | 0.79 | 0.74 | 0.36 | 0.80 |  | 0.80 |
|  | 0.523 | 0.732 | 0.107 | 0.711 | 0.511 | 0.560 | 0.697 | 0.098 | 0.888 | 0.062 | 0.289 | 0.723 |  | 0.906 |
| RM | 0.72 | 0.75 | 0.78 | 0.59 | 0.75 | 0.71 | 0.66 | 0.55 | 0.64 | 0.85 | 0.22 | 0.23 | 0.83 |  |
|  | 0.213 | 0.264 | 0.421 | 0.161 | 0.230 | 0.075 | 0.047 | 0.522 | 0.123 | 0.427 | 0.232 | 1.000 | 0.906 |  |

Grey half (bottom left) gives muscle coordination for arm elevation and the white half (top right) for arm depression. PCC – Pearson correlation coefficient. The p-values given report a paired samples t-test comparing phases; significant comparisons (p<0.050) in bold;
